# Factors Associated with *Leishmania* Asymptomatic Infection: Results from a Cross-Sectional Survey in Highland Northern Ethiopia

**DOI:** 10.1371/journal.pntd.0001813

**Published:** 2012-09-27

**Authors:** Estefanía Custodio, Endalamaw Gadisa, Luis Sordo, Israel Cruz, Javier Moreno, Javier Nieto, Carmen Chicharro, Abraham Aseffa, Zelalem Abraham, Tsegaye Hailu, Carmen Cañavate

**Affiliations:** 1 Centro Nacional de Medicina Tropical, Instituto de Salud Carlos III, Madrid, Spain; 2 Armauer Hansen Research Institute, Addis Ababa, Ethiopia; 3 Centro Nacional de Epidemiología, Instituto de Salud Carlos III, Madrid, Spain; 4 CIBER en Epidemiología y Salud Pública, Granada, Spain; 5 WHO Collaborating Center for Leishmaniasis, Servicio de Parasitología, Centro Nacional de Microbiología, Instituto de Salud Carlos III, Madrid, Spain; 6 Amhara Regional State Laboratory, Bahir Dar, Ethiopia; National Institutes of Health, United States of America

## Abstract

**Background:**

In northern Ethiopia the prevalence of visceral leishmaniasis is steadily rising posing an increasing public health concern. In order to develop effective control strategies on the transmission of the disease it is important to generate knowledge on the epidemiological determinants of the infection.

**Methodology/Principal Findings:**

We conducted a cross-sectional survey on children 4–15 years of age using a multi staged stratified cluster sampling on high incidence sub-districts of Amhara regional state, Ethiopia. The survey included a socio-demographic, health and dietary questionnaire, and anthropometric measurements. We performed rK39-ICT and DAT serological tests in order to detect anti-*Leishmania* antibodies and carried out Leishmanin Skin Test (LST) using *L.major* antigen. Logistic regression models were used. Of the 565 children surveyed 56 children were positive to infection (9.9%). The individual variables that showed a positive association with infection were increasing age, being male and sleeping outside [adjusted odds ratios (95% CI): 1.15 (1.03, 1.29), 2.56 (1.19, 5.48) and 2.21 (1.03, 4.71) respectively] and in relation to the household: past history of VL in the family, living in a straw roofed house and if the family owned sheep [adjusted OR (95% CI): 2.92 (1.25, 6.81), 2.71 (1.21, 6.07) and 4.16 (1.41, 12.31) respectively].

**Conclusions/Significance:**

A behavioural pattern like sleeping outside is determinant in the transmission of the infection in this area. Protective measures should be implemented against this identified risk activity. Results also suggest a geographical clustering and a household focalization of the infection. The behaviour of the vector in the area needs to be clarified in order to establish the role of domestic animals and house materials in the transmission of the infection.

## Author Summary

Visceral leishmaniasis is a vector borne disease that can be fatal if left untreated. Its prevalence is steadily rising in northern Ethiopia posing a public health challenge in the region. We conducted a study on the factors associated to asymptomatic infection in Libo Kemkem and Fogera, Amhara regional state, where little is known about *Leishmania* transmission. Sleeping outside was identified as a risk activity so measures towards it are recommended. Our results also showed a geographical clustering and a household focalization of the infection although the reasons behind it are not clearly understood. More entomological studies are needed in order to clarify the vecto's behaviour in the area. Individuals living in houses that owned sheep were more likely to be infected but no association was found with other domestic animals like cattle chicken or dogs. These results add up to the debate found in the literature regarding the role of domestic animals in the transmission of *Leishmania* in different regions of the world. No specific recommendation should be given until the exact role of the domestic animal in the transmission cycle is clearly understood.

## Introduction

Visceral leishmaniasis (VL) or kala-azar is a neglected vector-borne parasitic disease that manifests with irregular bouts of fever, substantial weight loss, weakness, hepatosplenomegaly and pancytopenia, and that is fatal if left untreated [1]. It has an estimated annual incidence of 500 000 clinical cases with 50 000 associated deaths and 2 357 000 disability-adjusted life years lost [2]. It is mainly concentrated in few major foci and the East African *Leishmania donovani* focus is the second largest, with the highest incidence in Ethiopia and the Sudan [2].

VL is caused by protozoan parasites of the *L.donovani species* complex transmitted to human and animal hosts by the bite of phlebotomine sand flies. It has already been determined that large numbers of individuals in endemic areas are infected with the parasite but do not develop any signs or symptoms of the disease. The reported ratio of asymptomatic infections to VL clinical cases varies widely from 4∶1 in Kenya [3] to 50∶1 in Spain [4]. This variation is presumed to reflect differences in parasite virulence and host population characteristics, and may also depend on the study designs and on the tests used to define asymptomatic infection [1].

The methods more widely used in order to assess asymptomatic infection in the field are a) serological assays that detect anti-*Leishmania* antibodies based either on the direct agglutination test (DAT) or the rK39-immunochromatographic test (rK39-ICT) and b) Leishmanin Skin Test (LST) that measures cell-mediated immunity against *Leishmania* [5], [6].

It is important to generate knowledge on the factors associated with asymptomatic infection for the optimal design and implementation of prevention and control strategies of VL, as asymptomatically infected individuals can harbor latent parasite and may act as reservoirs for new infection or become ill if immunosuppression occurs [7], [8].

In northern Ethiopia, the prevalence of VL is steadily rising posing an increasing public health concern. The region has recently experienced epidemics in previously unaffected areas [2]. In 2005, a kala-azar outbreak occurred in the district of Libo Kemkem in Amhara regional state, described by Alvar et al [9]. A case control study was conducted there in 2007 to evaluate the risk factors associated with the clinical form of the disease [10].

As it has been previously stated, the epidemiological determinants of clinical VL and sub clinical infection are not necessarily the same [11] but both are of interest to better understand the transmission of the disease.

Thus, the aim of this study is to describe the factors associated with asymptomatic *Leishmania* infection among the villages with high incidence of VL in Libo Kemkem and Fogera in order to complement the already existing information on VL transmission in the area and help the Amhara regional health authorities to develop effective strategies to control the transmission of the disease.

## Materials and Methods

### Study area and population

**Figure 1:**
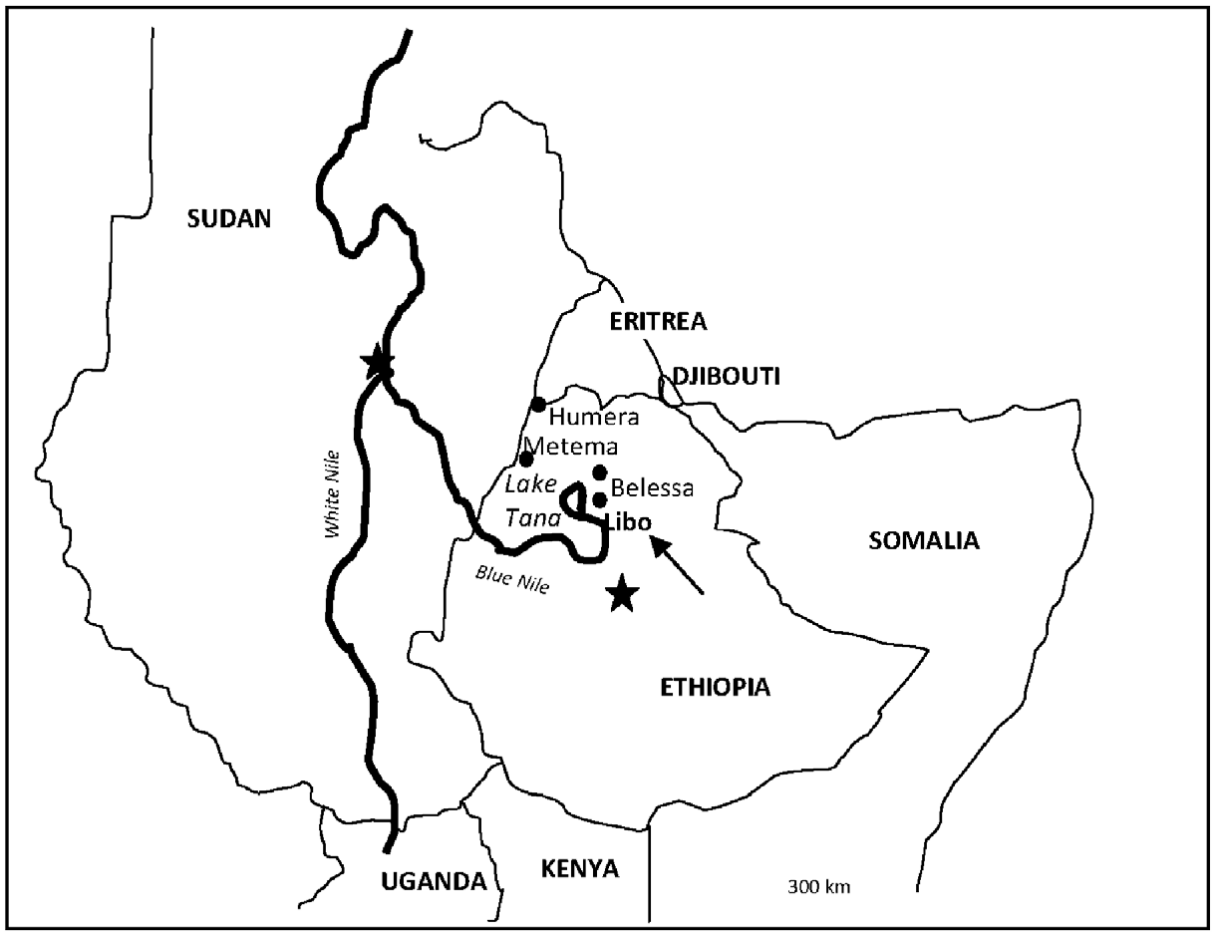
Location of the study area.

**Figure 2:**
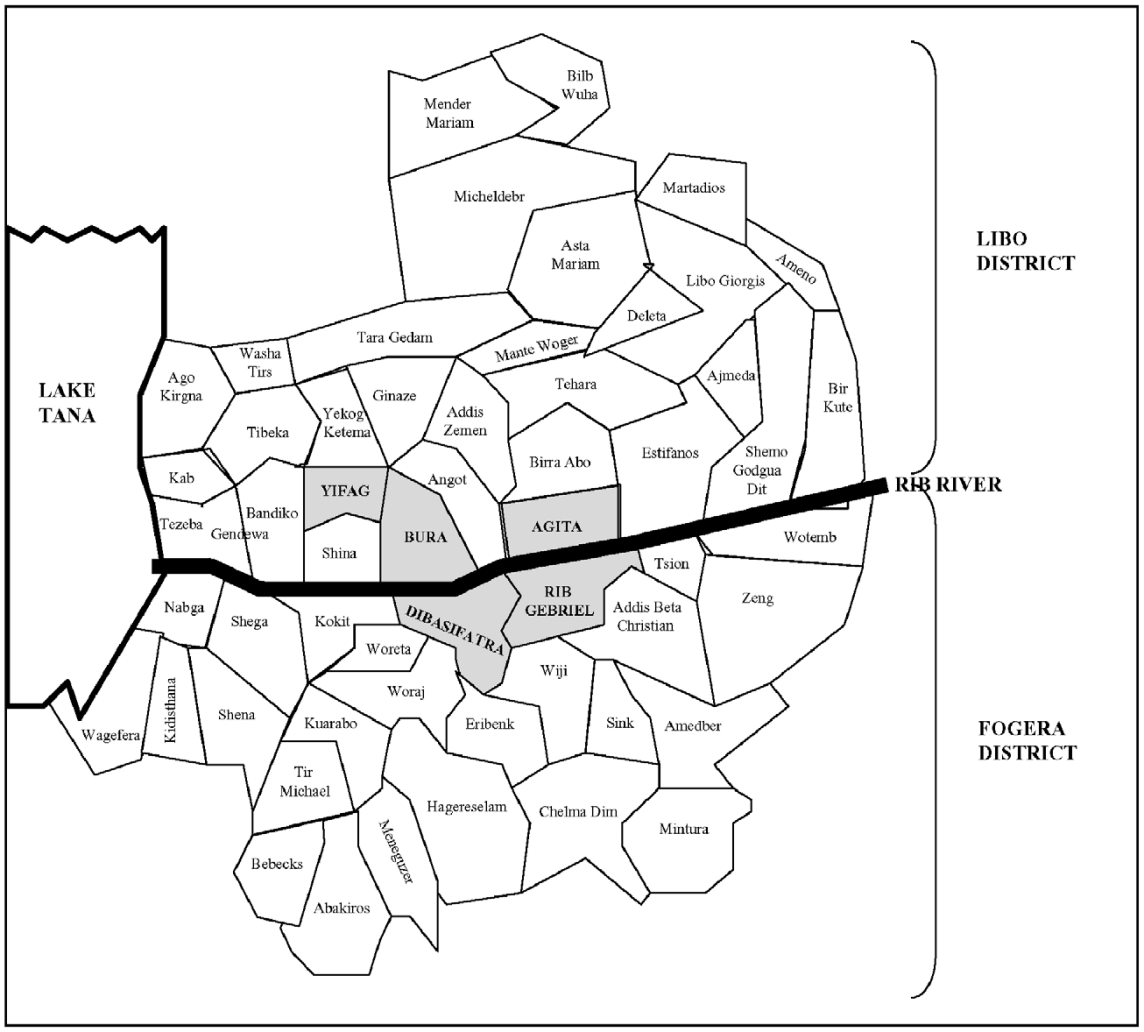
Location of the sub-districts on which the study was performed (grey background). Figures were adapted from Alvar et al. *Am. J. Trop. Med. Hyg.,* 77(2), 2007, pp. 275–282.

The study was conducted during May–July 2009 in the districts (*weredas*) of Libo Kemkem and Fogera (Amhara regional state, Ethiopia), see Figure 1 and Figure 2. These are adjacent districts most affected by the outbreak of VL that occurred in 2005 [9]. In 2009, the population numbered 198 374 and 226 595 in Libo Kemkem and Fogera, respectively. The economic status of the population is uniformly low. The districts are located in a black cotton clay soil flat plain (1800–2000 meters a.s.l.). Human activities related to intensive cultivation of *teff*, maize, beans, oilseeds, rice and cotton, have reduced the natural vegetation to scattered clumps of acacia trees. Most of the area is flooded during the rainy season (July–September) and dried up during the dry season (November–March), resulting in deep cracks in the soil surface, which could turn into breeding sites for the putative vector *Phlebotomus orientalis* [12], [13].

### Study design

The study was carried out within the framework of a UBS Optimus Foundation funded project called Visceral Leishmaniasis and Malnutrition in Amhara State, Ethiopia, which among its specific objectives aimed to characterize nutritional, immunological, and parasitological aspects of the school age children population in the districts of Fogera and Libo Kemkem. Sample size was calculated according to project goals using an expected malnutrition prevalence of 20% and applying a design effect of 2.

Population sampling was carried out by a multi-staged cluster survey. Primary sampling units were sub-districts (*kebeles*) with high incidence of VL according to the 2008 register of the Addis Zemen VL Treatment Centre: Bura, Yifag Akababi and Agita from Libo Kemkem and Sifatra and Rib Gebriel from Fogera. Secondary sampling units were randomly selected villages (*gotts*) in each of the selected sub-districts. Third sampling units were randomly selected households in each of the villages. All children with reported age between 4 and 15 years living in the selected household at the time of the survey, and with no previous history of VL were included in the study, as long as they were asymptomatic (absence of VL symptoms: fever for >2 weeks, in combination with either enlargement of spleen and/or liver, or weight loss).

### Data collection

A blood sample was taken from the selected children in order to detect anti-*Leishmania* antibodies. The rK39-ICT (Kalazar Detect Rapid Test, InBios International Inc., USA) was performed following the manufacturers' instructions. DAT with freeze-dried antigen (ITMA-DAT/VL, Prince Leopold Institute of Tropical Medicine, Antwerp, Belgium) was performed on blood-impregnated filter paper following the screening method according to the manufacturer's protocol. Titers ≥1∶3200 were considered positive.

Leishmanin Skin Test was carried out using *L. major* antigen (Leishmanin batch 123-2; Pasteur Institute, Iran). The test was read 48 hours later by the ballpoint pen method. An induration with an average of two perpendiculars ≥5 mm was considered as positive.

All children were measured and weighed according to standard World Health Organization (WHO) procedures [14]. Wasting was defined as Body Mass Index (BMI) for age Z score (BAZ)<−2, and stunting as Height for Age Z score (HAZ)<−2 according to the 2006 WHO Growth Standards for children ≤5 years and to the 2007 WHO Growth Reference for children >5 years respectively [15].

Care providers of the children were interviewed by trained health professionals using standardized questionnaires that included questions on demographics, household characteristics, child health, dietary habits and VL prevention behaviours. The questionnaires used were pretested and translated into Amharic, the local language.

### Data Analysis

The primary outcome of interest was *Leishmania* “asymptomatic infection” defined as a positive result in rK39-ICT, DAT or LST and the absence of VL signs and symptoms (fever for >2 weeks, in combination with either enlargement of spleen and/or liver, or weight loss). The serological tests (rK39-ICT and DAT) and the LST measure different types of immune response and are thus not likely to produce the same results. Therefore we created two secondary outcomes: a) Seropositive: positive to rK39-ICT and/or DAT irrespective of the LST result and b) LST Positive: positive to LST irrespective of the serostatus.

We attempted to describe the factors associated with “asymptomatic infection” and then to differentiate the factors associated with the seropositivity and LST positivity by making independent analysis for the three outcomes described above.

Since more than one child was sampled per household, the non-independence of children from the same household had to be taken into account. Therefore, potential risk factors were evaluated by odds ratios (OR) using random effects logistic regression with households defined as the group variable. To describe the amount of aggregation existing in VL asymptomatic infection within household units, the percentage of explained variance attributed (rho) was estimated in the adjusted models. Socioeconomic, behavioural, nutritional and dietary variables were assessed in univariate and multivariate analysis (listed in Table S1 and Table S2). Variables associated with each of the outcomes of interest at the p<0.10 level in the univariate analysis (univariate random effects logistic regression) were included in the multivariate regression procedure (multivariate random effects logistic regression). The final model was obtained by using a manual backward stepwise procedure. Variables with a p-value ≤0.05 were retained in the model. Age and sex, considered biologically relevant, were kept in the model independently of their level of association. Final multivariate models included all variables for which adjusted estimates are presented. A p value less than 0.05 was considered statistically significant.

Data analysis was performed using AnthroPlus v1.02 (WHO, Geneva, Switzerland), SPSS version 18.0 (SPSS Inc., Chicago, Illinois, USA) and STATA version 11.0 (StataCorp LP, College Station, Texas, USA).

### Ethical considerations

The study was approved by the ethical advisory boards of Instituto de Salud Carlos III in Spain and the Armauer Hansen Research Institute and the Ethiopian National Ethical Review Committee in Ethiopia. Support letters were obtained from the Amhara regional state and the district Health Bureaus. All parents/guardians gave written informed consent prior to the enrolment of their children in the study. Assent was also obtained from children ≥11 years of age.

## Results

A total of 639 children were screened, 30 children were excluded because they reported having had VL in the past (28) or their VL history was unknown (2). Forty-four children reported symptoms compatible with VL (fever for >2 weeks, in combination with either enlargement of spleen and/or liver, or weight loss) and were also excluded from the analysis. This resulted in a final sample of 565 children eligible for the study.

All the villages selected were rural. Two hundred and seventy eight households were surveyed. Around 90% of them were headed by males and in more than 99% the occupation of the head was related to farming activities (farmer, labourer, cotton worker, etc.). Ninety eight per cent of the households reported owning land. The mean size of land owned by a household was 1.6 Ha (range 0.01–8 Ha). Only 6.4% of the households owned more than 3 Ha. More than 95% of the households reported owning some type of domestic animals, mainly cows (89.9%), chicken (58.8%) and sheep (23.5%). Thirty three per cent of the households had radio and only 0.4% had access to electricity.

Of the 565 children surveyed, 51.1% were boys and 48.9% were girls and the mean age was 8.8 (3.2 SD) years. Fifty six children (9.9%) had asymptomatic infection, of which 35 (6.2%) were seropositive [30 (5.3%) positive to DAT and 8 (1.4%) positive to rK39-ICT)] and 30 (5.5%) were LST positive. There was a wide variation in the number of asymptomatically infected children according to villages, with the villages in Bura *kebele* presenting the highest frequencies (see Table 1).

**Table 1:** Asymptomatic infection, seropositivity and LST positivity prevalence by gott.

				Asymptomatic infection*		Seropositive*			LST positive* †	
Name of cluster/Gott	Kebele	Woreda	N	n	%	n	%	N	n	%
Fogerie Mender	Agita	Libo Kemkem	29	0	0	0	0	29	0	0
Fuat Fuat	Agita	Libo Kemkem	29	2	6.9	2	6.9	26	0	0
Gilgel Terara	Agita	Libo Kemkem	32	0	0	0	0	29	0	0
Melagud	Agita	Libo Kemkem	28	0	0	0	0	28	0	0
Medroge	Bura	Libo Kemkem	42	9	21.4	8	19	42	6	14.3
Mehal-Egziabherab	Bura	Libo Kemkem	28	9	32.14	1	3.6	28	8	28.6
Menta-Warka	Bura	Libo Kemkem	26	8	30.7	6	23.1	26	2	7.7
Quara	Bura	Libo Kemkem	32	5	15.6	1	3.1	31	4	12.9
Gultoch	D. Sifatra	Fogera	31	3	9.7	2	6.4	31	2	6.4
Lahada	D. Sifatra	Fogera	33	2	6.1	1	3	32	1	3.1
Ras Diba	D. Sifatra	Fogera	33	0	0	0	0	32	0	0
Sifatra	D. Sifatra	Fogera	28	4	14.3	3	10.7	25	1	4
Amaga	Rib Gebriel	Fogera	30	3	10	3	10	30	0	0
Denboch	Rib Gebriel	Fogera	35	2	5.7	1	2.9	35	1	2.9
Gichoch	Rib Gebriel	Fogera	35	2	5.7	2	5.7	32	2	6.2
Gombel	Rib Gebriel	Fogera	30	4	13.3	2	6.7	30	3	10
Ansha	Yifag Akababi	Libo Kemkem	31	2	6.4	2	6.4	31	0	0
Bata	Yifag Akababi	Libo Kemkem	33	1	3	1	3	32	0	0
**Total**			565	56	9.9	35	6.2	549	30	5.5

* As defined in M&M section.

† Total number of children with LST performed was 549, % calculated according to that number.

Among the children, 223 (39.7%) were found to be stunted and 119 (21.2%) were wasted. Only 5% had consumed animal food source products the day before the interview.

### Unadjusted analysis of infection

Table 2 and Table 3 summarize the individual and household characteristics that showed significant association in the univariate analysis with “asymptomatic infection”, seropositivity and LST positivity as defined in the Material and Methods section.

**Table 2:** Individual variables associated with asymptomatic infection*. Unadjusted analysis†.

		Asymptomatic infection		Seropositivity		LST positivity§	
Factor	N (%)	Positive n (%)	Odds ratio (95% CI)	Positive n (%)	Odds ratio (95% CI)	Positive n (%)	Odds ratio (95% CI)
**Child age**							
Years	565 (100)	56 (9.9)	1.21 (1.07, 1.36)	35 (6.2)	1.15 (1.01, 1.31)	30 (5.5)	1.24 (1.07, 1.44)
*p*			0.002		0.03		0.004
**Child sex**							
Girl	276 (48.9)	16 (5.8)	Reference	9 (3.26)	Reference	9 (3.4)	Reference
Boy	289(51.1)	40 (13.8)	3.36 (1.55, 7.28)	26 (9.0)	3.86 (1.46, 10.24)	21 (7.5)	2.66 (1.06, 6.67)
*p*			0.002		0.007		0.04
**Body Mass Index for Age**							
Z scores	562 (99.5)	56 (9.9)	0.68 (0.48, 0.98)	35 (6.2)	0.70 (0.46, 1.07)	30 (5.5)	0.75 (0.49, 1.14)
*p*			0.04		0.1		0.18
**Child sleeps outside**							
No	343 (60.8)	20 (5.8)	Reference	16 (4.6)	Reference	7 (2.1)	Reference
Yes	221 (39.2)	36 (16.3)	3.46 (1.65, 7.22)	19 (8.6)	1.69 (0.74, 3.88)	23 (10.7)	6.50 (2.25, 18.73)
*p*			0.001		0.21		0.001
**Child herds the cattle**							
No	227 (41.4)	11 (4.7)	Reference	9 (3.8)	Reference	3 (1.3)	Reference
Yes	322 (58.7)	45 (13.6)	4.30 (1.80, 10.27)	26 (7.9)	2.48 (0.96, 6.40)	27 (8.4)	8.25 (2.20, 31.0)
*p*			0.001		0.06		0.002
**Child uses bed net**							
No	317 (59.8)	38 (11.3)	Reference	20 (5.9)	Reference	23 (7.0)	Reference
Yes	213 (40.2)	18 (7.9)	0.63 (0.30, 1.33)	15 (6.6)	1.12 (0.48, 2.60)	7 (3.2)	0.36 (0.13, 1.00)
*p*			0.23		0.79		0.05
**Overall**	565	56 (9.9)		35 (6.2)		30 (5.5)	

* As defined in the Material and Methods section.

LST = Leishmanin Skin Test.

† Odds ratios obtained by univariate random effects logistic regression.

§ Total number of children with LST performed was 549, % calculated according to that number.

**Table 3:** Household characteristics associated with asymptomatic infection*. Unadjusted analysis†.

		Asymptomatic infection		Seropositivity		LST positivity§	
Factor	N (%)	Positive n (%)	Odds ratio (95% CI)	Positive n (%)	Odds ratio (95% CI)	Positive n (%)	Odds ratio (95% CI)
**People in the family**							
Persons	604 (99.8)	56 (9.9)	1.28 (1.00, 1.62)	35 (6.2)	1.17 (0.90, 1.54)	30 (5.5)	1.31 (0.98, 1.75)
*p*			0.04		0.24		0.07
**Past history of kala azar in the family**							
No	415 (73.6)	33 (8.0)	Reference	18 (4.3)	Reference	21 (5.2)	Reference
Yes	149 (26.4)	23 (15.4)	2.99 (1.28, 7.00)	17 (11.4)	4.13 (1.53, 11.17)	9 (6.2)	1.66 (0.66, 4.14)
*p*			0.01		0.005		0.48
**Household own sheep**							
No	502 (88.8)	43 (8.6)	Reference	27 (5.4)	Reference	23 (4.7)	Reference
Yes	63 (11.2)	13 (20.6)	3.33 (1.19, 9.32)	8 (12.7)	2.92 (0.67, 3.72)	7 (11.3)	2.64 (0.83, 8.33)
*p*			0.02		0.07		0.09
**Overall**	565	56 (9.9)		35 (6.2)		30 (5.5)	

* As defined in the Material and Methods section.

LST = Leishmanin Skin Test.

† Odds ratios obtained by univariate random effects logistic regression.

§ Total number of children with LST performed was 549, % calculated according to that number.

The individual factors that showed a positive association with “asymptomatic infection” were: increasing age, male sex, sleeping outside, cattle herding and decreasing BAZ. In relation to household conditions an increasing number of people living in the household, having a past history of VL in the family, and owning sheep showed a direct and significant association. Living in a house with straw roof versus corrugated iron roof showed a direct association close to significance (1.92 [0.92, 4.03], p = 0.08).

Increasing age and being male were the only two individual variables that showed a positive and significant association with seropositivity. Cattle herding showed a positive and close to significance relationship (p = 0.06). In terms of household variables only if someone in the family had had VL in the past was directly and significantly associated with it. Owning sheep showed a positive but not significant association (p = 0.07).

The individual variables that showed a direct association with LST positivity were the same as those for “asymptomatic infection”, except for BAZ. The use of bed net by a child, although not statistically significant suggested an inverse association (p = 0.050). In terms of household conditions, increasing number of people in the family and owning sheep showed a positive but not significant association (p = 0.07 and p = 0.09 respectively).

### Adjusted analysis

Table 4 shows the results of the multivariate logistic regression for “asymptomatic infection”, seropositivity” and LST positivity.

**Table 4:** Factors associated with Leishmania asymptomatic infection* in children. Adjusted analysis†.

	Asymptomatic infection	Seropositivity	LST positivity
Factor	Odds ratio (95% CI)	Odds ratio (95% CI)	Odds ratio (95% CI)
**Child age**			
Years	1.15 (1.03, 1.29)	1.14 (1.00, 1.30)	1.19 (1.02, 1.40)
*p*	0.02	0.07	0.03
**Child sex**			
Girl	Reference	Reference	Reference
Boy	2.56 (1.19, 5.48)	3.55 (1.31, 9.63)	1.88 (0.70, 4.94)
*p*	0.02	0.01	0.2
**Child sleeps outside**			
No	Reference	″	Reference
Yes	2.21 (1.03, 4.71)		5.51(1.77, 17.20)
*p*	0.04		0.003
**History of past kala azar in family**			
No	Reference	Reference	
Yes	2.92 (1.25, 6.81)	4.67 (1.59, 13.75)	″
*p*	0.01	0.005	
**Household roof material**			
Corrugated iron	Reference		
Straw	2.71 (1.21, 6.07)	″	″
*p*	0.02		
**Household owned sheep 3 years before and at the time of the survey**			
No	Reference	″	″
Yes	4.16 (1.41, 12.31)		
*p*	0.01		

* As defined in the Material and Methods section.

† Odds Ratios obtained by multivariate random effects logistic regression.

LST = Leishmanin Skin Test.

″Variable not included in the final model for the outcome of interest.

The individual variables that kept in the model positively associated with “asymptomatic infection” after adjustment were: increasing age (per year), being male and sleeping outside at any time of the year [OR (95% CI): 1.15 (1.03, 1.29), 2.56 (1.19, 5.48) and 2.21 (1.03, 4.71) respectively]. The household characteristics that remained positively associated with this same outcome after adjustment were: past history of VL in the family, living in a straw roofed house and if the family owned sheep [OR (95% CI): 2.92 (1.25, 6.81), 2.71 (1.21, 6.07) and 4.16 (1.41, 12.31) respectively].

Being male and past history of VL in the family were the only variables that kept direct and significant association with seropositivity after adjustment [OR (95% CI): 3.55 (1.31, 9.63), and 4.67 (1.59, 13.75) respectively].

And increasing age and sleeping outside were the only factors positively and significantly associated with LST after adjustment [OR (95% CI): 1.19 (1.02, 1.40) and 5.51 (1.77, 17.20) respectively].

A significant level of aggregation within household units was found for the three outcomes analyzed, being strongest in the case of seropositivity. “Asymptomatic infection” (rho = 32%, 95%CI: 11 to 65, p = 0.012), seropositivity (rho = 44%, 95% CI: 18 to 73, p = 0.004) and LST positivity (rho = 40%, 95% CI: 13 to 76, p = 0.022).

No significant association was found between any of the outcomes analysed and stunting; the number of meals consumed or consumption of animal source food products by the child the day before the survey; number of children in the household; age, sex, or education of the head of the household; wall construction material and condition, household electricity, radio or land owning, the existence of an animal shed, animal dung or a termite mound near the house, if the household owned dogs, cattle or chicken, the number of cattle, chicken or sheep owned by the house; the number of bed nets in the household or the house spraying status.

## Discussion

The prevalence of asymptomatic infection found in our study sample as well as the factors associated with it, differed depending on the outcome variable used for the analysis.

The discordances observed between serology and LST have been discussed elsewhere [7], [16]–[18]. The last LST screening in the area was conducted in 2005 as part of the outbreak assessment, and the prevalence of LST positivity was considerably higher than in our study, 34% for men and 26% for women [9]. The differences in design may account for this marked difference, as the study by Alvar et al was carried out in three villages reported to be highly affected (all belonging to Bura sub-district) and a fourth one selected from Shina sub-district but only a few kilometres away from Bura. Also, the cited study was conducted in a population with a different age distribution (age range; 0.7–60 years) with more than 50% of the sample being 15 years or older. Seventy per cent of the LST positive cases found in that study belonged to this older age group. Finally, the treatment interventions carried out in the area in the time period between the two studies could have reduced the transmission. A similar observation, a reduction of LST positivity in one year period from 30.1% to 17.3%, has been documented in an *L.donovani* focus of south Ethiopia [17].

The strong variation in the prevalence of asymptomatic infection among clusters highly endemic for VL is congruent with the spatial clustering observed in other studies of asymptomatic infection [19], [20] and of clinical VL cases [21], [22]. Notably, Bura, the kebele where the 2005 outbreak started, has maintained the highest prevalence ever since [9].

The increase in asymptomatic infection rate with age observed in our study area is also consistent with an endemic focus of VL, in spite of the low VL incidence situation reached after the outbreak [23], [24]. The permanence of LST reactivity is thought to be a consequence of cumulative past exposure, thus prevalence typically rises with age [25]. The positive association between *Leishmania* infection and older age, as well as with male sex, has also been related to activities like cattle herding or sleeping outside, that imply an increased potential exposure to the sand fly vector, and that are culturally specific to male adolescents and male adults [19], [26]. Our results would support this hypothesis, as cattle herding and sleeping outside were also identified in our study population as risk activities for asymptomatic infection and had previously been identified as risk factors for VL in South Ethiopia [27] and North Ethiopia (in our study area) as well [10]. The greater exposure to sand flies when herding livestock can be associated with the staying outside at dusk and dawn when the sand flies are supposed to be active [28] and also with an increased proximity to acacia trees. *Acacia*-*Balanites* forest growing on black cotton soils have been described as specific habitats with abundance of *Phlebotomus orientalis* [29]. Other studies have described the risk for humans to contract the infection by intruding into this type of environment [30]. Resting under acacia trees was identified as a risk factor for VL in our study area [10] and among our surveyed population 82% of the herder children reported resting under acacia trees while herding. However it is important to highlight that in the adjusted analysis only sleeping outside remained significant suggesting that this behaviour is associated with infection independently of the cattle herding activity.

Poor nutritional status has been associated with a higher risk of developing visceral leishmaniasis in other studies [31]–[34] although to the best of our knowledge, an association with asymptomatic infection has not yet been described. In our findings a better nutritional status (increasing BAZ) appeared as protector for asymptomatic infection but only in the unadjusted analysis, so we can not conclude there is association between nutritional status, measured by anthropometry, and asymptomatic infection in our study population.

The use of bed net appeared to be protective for LST positivity and the global “asymptomatic infection” outcome, but did not reach a significant association, which is in agreement with other studies in relation to asymptomatic infection [16], [35]. The protective effect of bed net use towards visceral leishmaniasis remains unclear, with variable results depending on the setting and study [11], [36]. The net conditions, nature of utilization and impregnation status were not assessed in our survey, and this can account for the lack of statistical significance found in our results.

A previous case of VL within the household appeared strongly associated with seropositivity and maintained the association with the global “asymptomatic infection” outcome. Other studies have also identified living close to a previous case of VL as a risk factor for *L.donovani* asymptomatic infection [3] or for the clinical form of the disease [35], [37], [38] suggesting the importance of the house as a micro focus in the spread of the disease. The significant amount of aggregation within the households of the three outcomes analysed in this study would further support this hypothesis. This may be related to the ecologic location of the household, although there have been studies that have failed to relate house or surrounding ecological characteristics with it [3], [39]. This house focalization may also be related to genetics or to the possibility of a domestic or peri-domestic transmission. It is important to highlight that the increased likelihood of asymptomatic infection among children with a past VL case in the family remained significant only for seropositivity and not for LST positivity, in concordance with findings of Bern et al in Bangladesh [35]. In one study conducted in Kenya, it was found that the association between LST positivity and previous VL cases in the family was significant only for women and young children, suggesting that women were exposed in and around the house and males, in addition, exposed elsewhere [40]. We tested this hypothesis by conducting separate analyses for male and female populations but results did not vary (data not shown). The theory of transmission within the household is only a supposition as the existence of a domestic reservoir has not yet been well substantiated in Ethiopia. Furthermore, most reports regarding *P.orientalis* point out that the vector is rarely encountered inside the houses [41], [42]. However in an entomological study conducted in eastern Sudan, it was reported that 75% of the *P.orientalis* captured were found indoors [43] indicating that some populations of the vector are more adapted to domestic habitats. This might be due to variations in construction materials used in building houses or to other microclimatic conditions [29]. The high altitude (1800–2000 mts) of Libo Kemkem and Fogera makes the ecoclimatic conditions rather unique for what is known about sand fly ecology. Therefore there is an urgent need to identify and understand the behaviour of sand flies in the area in order to come up with consistent conclusions.

Living in a straw roofed house versus an iron thatched one was the only house characteristic associated with asymptomatic infection. It could be related to socioeconomic status or to the potential of straw roofs to provide resting places for the sand fly that would increase its survival and abundance. Mud-type houses have been identified as risk factors for VL or asymptomatic infection before and have been associated with better living conditions or with the vector preference for mud crack walls for breeding and resting [22], [27], [38], [44]. However, regarding *P.orientalis* more studies are needed, as the few extant studies in the literature point out to an exophagic behaviour of the vector, ill suited with this hypothesis [41].

In relation to domestic animals the only significant association found was with owning sheep. The positive correlation of disease and the presence of sheep has already been described [19], [22], and has been explained by the greater biomass and the accumulation of animal dung that may be attractive to the sand flies, drawing the vectors into closer association with humans. However, the number of sheep, the presence of other livestock, or animal dung near the house did not show a significant association in our study. On the other hand, sheep herding can be associated to an increased contact to Acacia trees due to the fact that sheep are fed on Acacia fruits and leaves [45]. Our personal observation during surveys was that herders shake Acacia trees in order to make fruits fall, thus disturbing the suspected vector habitat and increasing the probabilities of being bitten.

One important limitation of our study is its cross sectional nature, which limits the making of causal inferences between the analysed factors and the infection. However, we believe the results are of interest in order to contribute to the existing knowledge in the area, and in order to support future analytical studies.

Our conclusion is that sleeping outside and selected housing factors were associated with higher rates of asymptomatic infection and our recommendation is that the behaviour of *P.orientalis,* the putative vector in the area should be further studied in order to clarify the role of domestic animals in the transmission cycle and in order to propose possible entomological interventions.

## Supporting Information

Checklist S1 **STROBE checklist.**
(DOC)

Table S1 **List of individual variables introduced in the univariate analysis.**
(DOC)

Table S2 **List of households variables introduced in the univariate analysis.**
(DOC)
