# Supplementary material for: Factors Associated with Leishmania Asymptomatic Infection: Results from a Cross-Sectional Survey in Highland Northern Ethiopia
Source: PLoS Negl Trop Dis. 2012 Sep 27;6(9):e1813. doi: 10.1371/journal.pntd.0001813 (PMC3459849; doi:10.1371/journal.pntd.0001813)
Supplement: Table S1 — List of individual variables introduced in the univariate analysis. (DOC) [file pntd.0001813.s002.doc]

| **Table S1: List of individual variables* introduced in the univariate analysis** | |
| --- | --- |
| **Variable** | **Categories** |
| **Biological** |  |
| Child age (years)* |  |
| Child sex (male/female) | Male |
|  | Female |
| **Behavioural** |  |
| Child sleeps outside (yes/no) | Yes |
|  | No |
| Child herds the cattle (yes/no) | Yes |
|  | No |
| Child uses bed net (yes/no) | Yes |
|  | No |
| **Nutritional** |  |
| Height for Age Z scores (HAZ)* |  |
| Moderate stunting defined as:-3<HAZ<-2 (yes/no) | Yes |
|  | No |
| Severe stunting defined as: HAZ < -3 | Yes |
|  | No |
| Body Mass Index for Age Z score (BAZ)* |  |
| Moderate wasting defined as:-3 <BAZ <-2 | Yes |
|  | No |
| Severe wasting defined as BAZ < -3 | Yes |
|  | No |
| **Dietary** |  |
| Number of meals consumed by the child the day before* |  |
| Child consumed any animal source food product the day before the survey | Yes |
|  | No |
| *Variables introduced in the analysis as continuous | |
