# Supplementary material for: Factors Associated with Leishmania Asymptomatic Infection: Results from a Cross-Sectional Survey in Highland Northern Ethiopia
Source: PLoS Negl Trop Dis. 2012 Sep 27;6(9):e1813. doi: 10.1371/journal.pntd.0001813 (PMC3459849; doi:10.1371/journal.pntd.0001813)
Supplement: Table S2 — List of households variables introduced in the univariate analysis. (DOC) [file pntd.0001813.s003.doc]

| **Table S2: List of household variables* introduced in the univariate analysis** | |
| --- | --- |
| **Variable** | **Categories** |
| **Demographic** |  |
| Number of people living in the household* |  |
|  |  |
| Number of children in the house* |  |
| Age of head of the household* |  |
| Sex of head of the household | Male |
|  | Female |
| Education of head of the household | No school |
|  | Primary school |
|  | Above primary school |
| **House material and household appliances** |  |
| Roof material | Straw |
|  | Corrugated iron |
| Wall material | Mud |
|  | Wood |
| Wall condition | No cracks |
|  | Some cracks |
|  | Cracks in almost all walls |
| Electricity | Yes |
|  | No |
| Radio | Yes |
|  | No |
| Has. of land owned by the house* | Less than 1 Ha. |
|  | 1-3 Has. |
|  | > 3 Has. |
| **Livestock and domestic animals** |  |
| Animal shed in the household | Yes |
|  | No |
| Distance to animal shed from the household (mts)* |  |
| Animal dung near the household | Yes |
|  | No |
| Household owns cattle in the previous three years and at the time of the survey | Yes |
|  | No |
| Number of cattle owned by the household at the time of the survey* |  |
| Household owns chicken in the previous three years and at the time of the survey | Yes |
|  | No |
| Number of chicken owned by the household at the time of the survey* |  |
| Household owns sheep in the previous three years and at the time of the survey | Yes |
|  | No |
| Household owns dogs in the previous three years and at the time of the survey | Yes |
|  | No |
| **Preventive measures** |  |
| Number of bed nets in the household* |  |
| Household has been sprayed | Yes |
|  | No |
| Time since household was last sprayed | Before one year ago |
|  | More than one year ago |
|  | Never |
| **Other** |  |
| Termite mound near the household | Yes |
|  | No |
| *Variables introduced in the analysis as continuous. | |
